# Biological tuners to reshape the bile acid pool for therapeutic purposes in non-alcoholic fatty liver disease

**DOI:** 10.1042/CS20220697

**Published:** 2023-01-05

**Authors:** Justine Gillard, Isabelle A. Leclercq

**Affiliations:** Laboratory of Hepato‐Gastroenterology, Institute of Experimental and Clinical Research, Université catholique de Louvain, Brussels, Belgium

**Keywords:** bile acids, enterohepatic cycle, gut microbiota, non alcoholic fatty liver disease, non-alcoholic steatohepatitis

## Abstract

Bile acids synthesized within the hepatocytes are transformed by gut microorganisms and reabsorbed into the portal circulation. During their enterohepatic cycling, bile acids act as signaling molecules by interacting with receptors to regulate pathways involved in many physiological processes. The bile acid pool, composed of a variety of bile acid species, has been shown to be altered in diseases, hence contributing to disease pathogenesis. Thus, understanding the changes in bile acid pool size and composition in pathological processes will help to elaborate effective pharmacological treatments.

Five crucial steps along the enterohepatic cycle shape the bile acid pool size and composition, offering five possible targets for therapeutic intervention. In this review, we provide an insight on the strategies to modulate the bile acid pool, and then we discuss the potential benefits in non-alcoholic fatty liver disease.

## The enterohepatic cycle of bile acids

**Figure 1:**
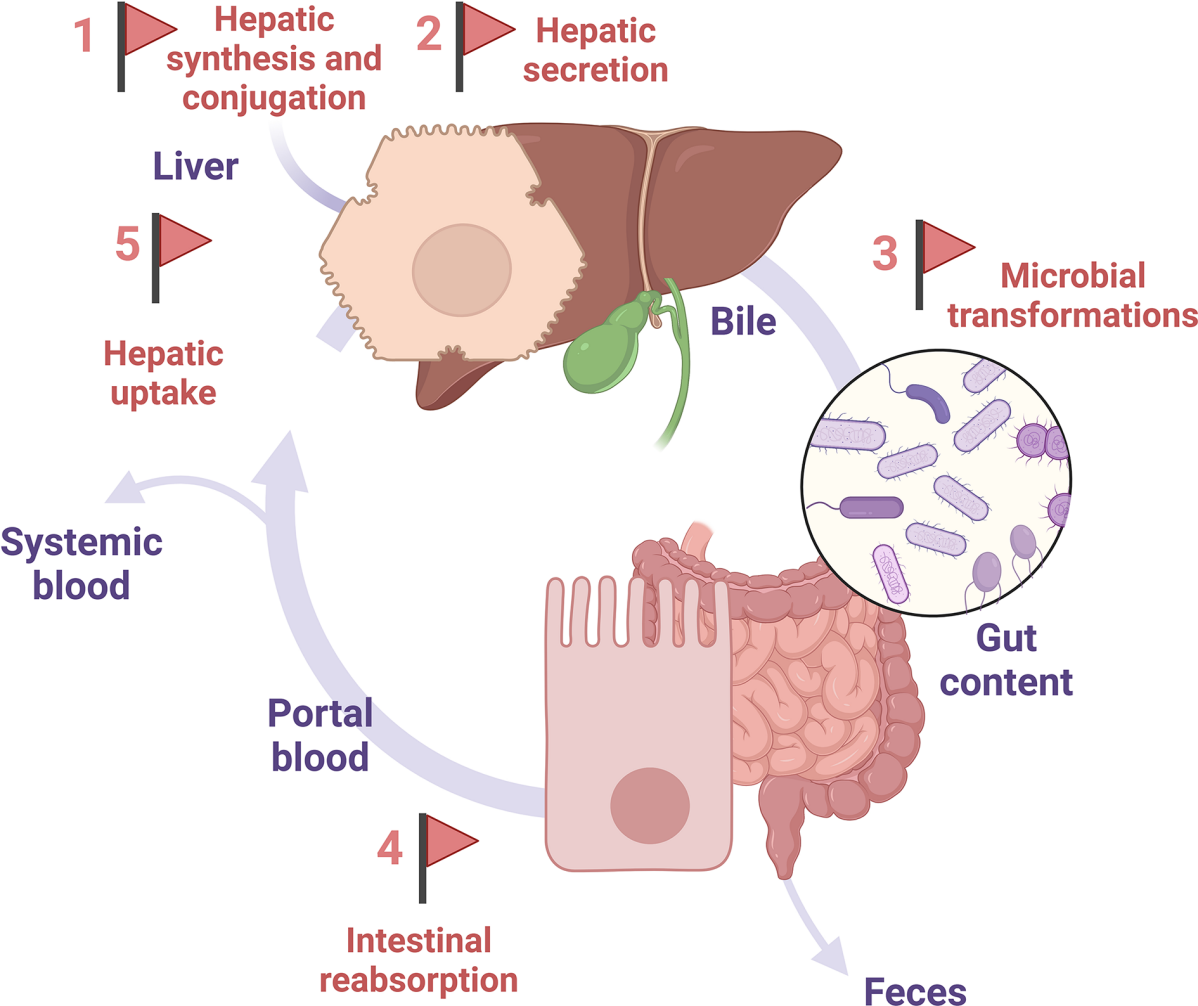
Five crucial steps along the enterohepatic cycle (represented by the flags) shape the bile acid pool size and composition Bile acids travel along the gut-liver axis in a perpetual and efficient cycle, with minute proportions that leak into the systemic circulation or are excreted in feces. In the hepatocytes, primary bile acids are synthesized from cholesterol (Flag 1). After conjugation to taurine or glycine, highly soluble conjugated bile acids are secreted at the biliary pole of the hepatocytes into the bile (Flag 2). Bile acids then reach the gut lumen, where they interact with a wide range of gut microorganisms and undergo transformations by microbial enzymes to yield secondary bile acids (Flag 3). The majority of bile acids are reabsorbed into the portal circulation (Flag 4) that carries them back to the liver where they are recaptured by the hepatocytes (Flag 5). These five steps along the enterohepatic cycle offer five possible targets for therapeutic interventions.

Most of the bile acids travel along the gut–liver axis in a perpetual cycle with minute proportions that leak into the systemic circulation or are excreted in feces. Five crucial steps along the enterohepatic cycle shape the bile acid pool size and composition (Figure 1).

**Figure 2:**
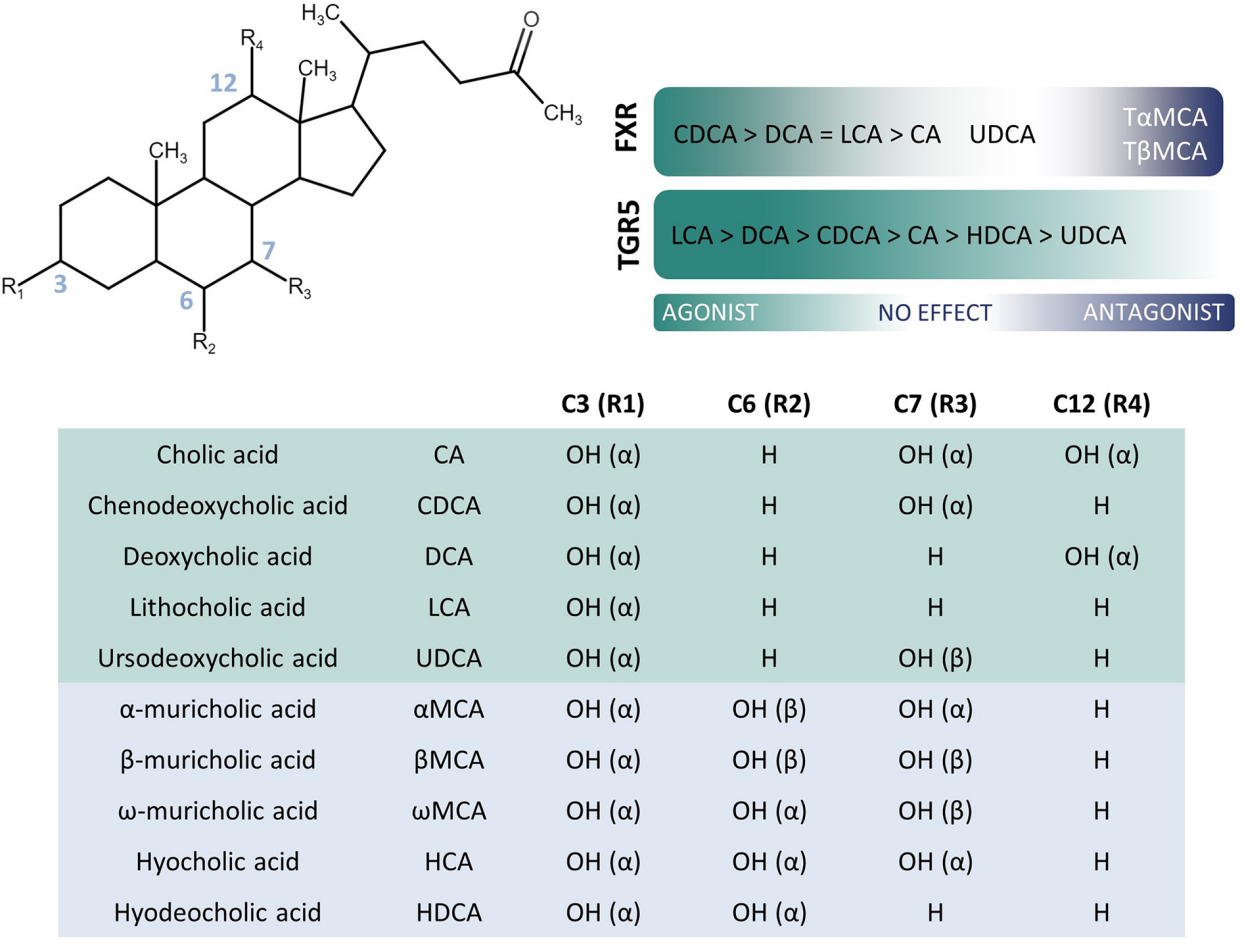
Bile acid structure and activity Summary of the main bile acid species, the moieties at C3 (R1), C6 (R2), C7 (R3) and C12 (R4) sites of the sterol backbone, and their agonistic and antagonistic activities for FXR and TGR5. In the table, the main bile acid species in humans are represented in green ant the additional murine bile acids in blue.

**Figure 3:**
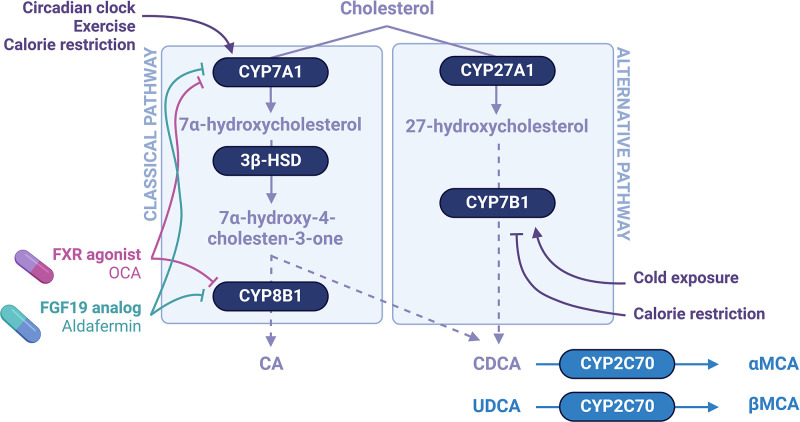
Bile acid synthesis through the classical and the alternative pathways The classical pathway is initiated by the rate-limiting CYP7A1 that converts cholesterol into 7α-hydroxycholesterol, further transformed into 7α-hydroxy-4-cholesten-3-one by the 3β-HSD. The CYP8B1 hydroxylates a proportion of 7α-hydroxy-4-cholesten-3-one to form CA. The rest is not hydroxylated by CYP8B1 and forms CDCA. The alternative pathway converts cholesterol into 27-hydroxycholesterol through the CYP27A1 and then the CYP7B1 hydroxylates it to form CDCA. Mouse but not human livers express CYP2C70 that hydroxylates CDCA and UDCA at the C6 position to generate α- and β-MCAs, respectively. The classical pathway for bile acid synthesis might be regulated by FXR agonists and FGF19 analogs. Some environmental factors also modulate bile acid synthesis through the classical and the alternative pathways.

The transformation of cholesterol into primary bile acids (Figure 2) requires the action of 17 hepatic enzymes involved in two pathways (the classical and the alternative pathways) [1]. The classical pathway producing cholic and chenodeoxycholic acids (CA and CDCA) is initiated by the rate-limiting cholesterol 7α-hydroxylase (CYP7A1) that converts cholesterol into 7α-hydroxycholesterol (Figure 3). The latter is further transformed into 7α-hydroxy-4-cholesten-3-one (called C4, a proxy for bile acid synthesis) by the 3β-hydroxysteroid dehydrogenase (3β-HSD). The sterol 12α-hydroxylase (CYP8B1) then hydroxylates a proportion of 7α-hydroxy-4-cholesten-3-one to form CA (Figures 2 and 3). The rest is not hydroxylated by CYP8B1 and forms CDCA (Figures 2 and 3). The alternative pathway converts cholesterol into 27-hydroxycholesterol through the sterol 27-hydroxylase (CYP27A1) and then the oxysterol 7α-hydroxylase (CYP7B1) hydroxylates it to form CDCA [2]. Some extrahepatic tissues do support the production of oxysterols but the reaction that converts oxysterols into CDCA is restricted to hepatocytes. In physiological conditions, the classical pathway generates 90–95% of bile acids and the alternative pathway 5–10% [3].

**Figure 4:**
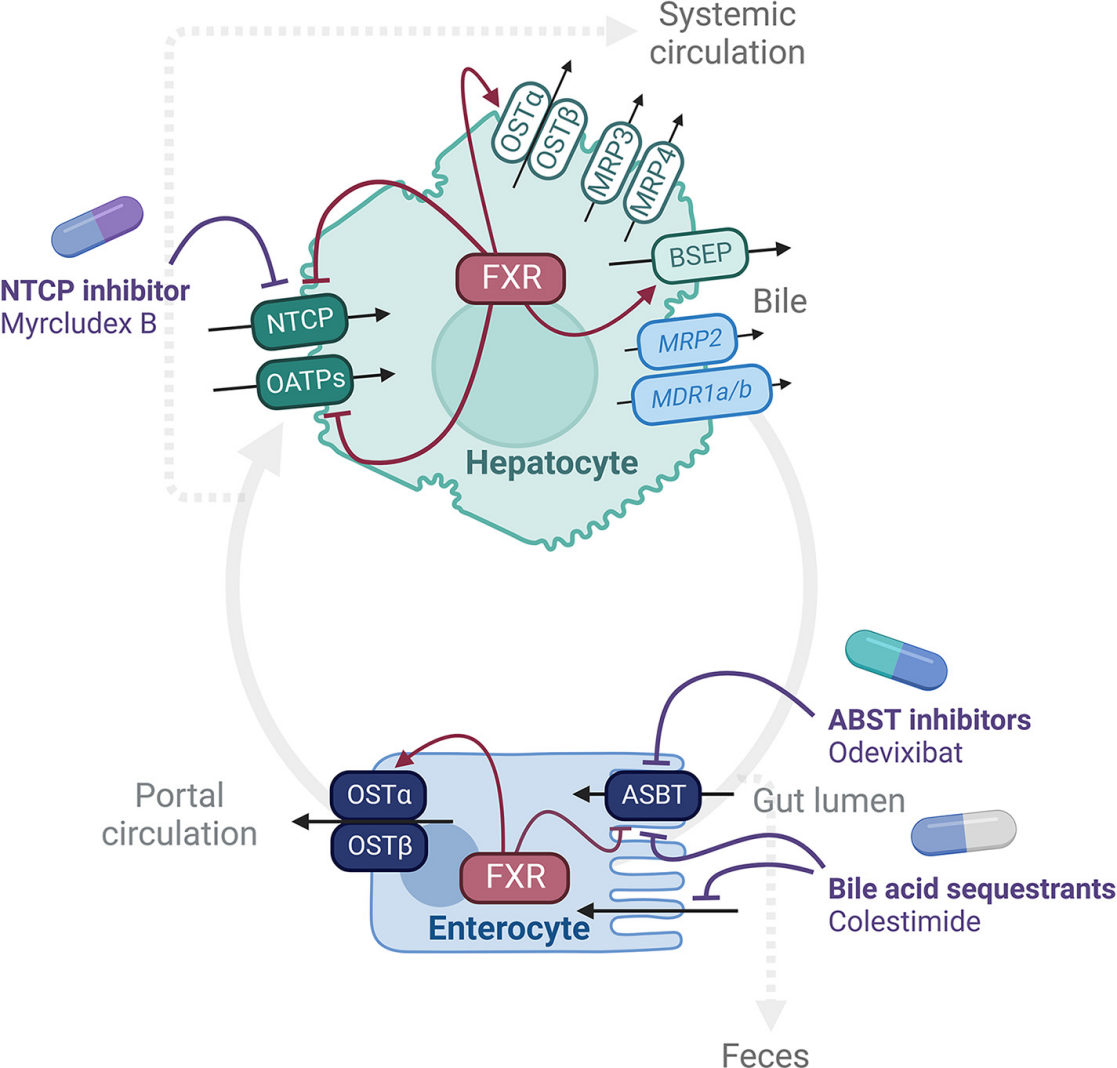
Bile acid transport in hepatocytes and enterocytes Bile acids are exported across the canalicular membrane of the hepatocytes through BSEP. In mice, MDR1a/1b and MRP2 additionally transport bile acids. Basolateral efflux of bile acids into the systemic circulation occurs through OSTα/β, MRP3 and MRP4. ASBT actively transports bile acids within the enterocytes of the terminal ileum, FABP6 delivers them to the basolateral membrane, and the OSTα/β heterodimer is required for transport across the membrane. Some bile acids also passively diffuse across the colonic epithelium to reach the portal circulation. The portal blood then carries the bile acids back to the liver where they are taken up in the hepatocytes by NTCP (which recaptures conjugated bile acids) and by OATP isoforms (which recaptures unconjugated bile acids). Bile acid transporters are under the regulation of FXR. Bile acids reabsorption and thus circulation are prevented by bile acid sequestrants and by inhibitors of transporters such as Myrcludex B for NTCP and Odevixibat for ABST.

Bile acids are conjugated to glycine or taurine under the action of the bile acid-coenzyme A synthase and the bile acid: amino acid transferase [1]. Highly soluble conjugated bile acids are then secreted out of the hepatocytes into the bile by the bile salt export pump (BSEP, Figure 4). Flowing along the bile canaliculi and bile ducts, bile directly reaches the duodenum or fills the gallbladder where it is concentrated and stored. Gallbladder contraction induced by food intake expulses bile into the duodenal lumen to facilitate emulsification, digestion and absorption of lipids.

In the intestinal lumen, bile acids interact with a wide range of microorganisms and undergo transformations by microbial enzymes to yield secondary bile acids (Figure 2). After the cleavage of glycine or taurine conjugates by the bile salt hydrolases (BSHs) [4], free CA and CDCA can be respectively converted into deoxycholic and lithocholic acids (DCA and LCA) by the multistep 7α-dehydroxylase reaction. Other microbial enzymes imprint other transformations to bile acids such as oxidation, epimerization or dehydroxylation [5].

Approximately 95% of bile acids present in the gut are reabsorbed into the portal circulation and 5% lost in feces [6]. While free secondary bile acids diffuse across the colonic epithelium, conjugated bile acids are actively reabsorbed through the enterocytes of the terminal ileum. The apical sodium-dependent bile salt transporter (ASBT) takes up bile acids, the fatty acid binding protein 6 (FABP6) delivers them to the basolateral membrane, and the heterodimer organic solute transporter α/β (OST α/β) is required for transport across the basolateral membrane (Figure 4).

The portal blood then carries the bile acids back to the liver where they are taken up in the hepatocytes [6]. The sodium taurocholate co-transporting polypeptide (NTCP) captures the conjugated bile acids and the organic anion transporting polypeptide (OATP) isoforms the unconjugated bile acids (Figure 4). This last step completes the effective enterohepatic cycle of bile acids. A small proportion of bile acids (approximately 5%) however escapes hepatic reuptake and spills out in the systemic circulation. In hepatocytes, bile acids from the digestive tract then mix with the newly synthesized ones.

## Mice as an experimental model to study bile acids

Most of the data on bile acids are acquired from murine models. However, the bile acid pools of mice and humans are substantially different (reviewed by Li and Dawson [7] and recapitulated in Figure 2). The murine liver produces primary CA and CDCA as well as ursodeoxycholic acid (UDCA) and further hydroxylates CDCA and UDCA at the C6 position using CYP2C70 to generate α- and β-muricholic acids (α- and β-MCAs), respectively (Figure 2) [8–10]. In turn, α- and β-MCAs are converted by the microbial enzymes to hyodeoxycholic acid (HDCA) and ωMCA. By contrast, CA and CDCA are the main primary bile acids in humans, while MCAs and HDCA are only present at very low concentrations in physiological conditions, as CYP2C70 is not expressed in human liver [7]. Also, bile acids are conjugated to glycine (75%) and to taurine (25%) in humans, while nearly exclusively to taurine in mice [7]. The gut microbial composition (and thus the capacity for bile acid transformation) also differs between humans and mice [11]. As a consequence, the bile acid pool in mice is more hydrophilic and more prone to be actively reabsorbed through bile acid transporters, while intestinal reabsorption in humans rather relies on passive diffusion [7]. Additionally, mouse, but not human, livers express CYP2A12, an enzyme that rehydroxylates secondary bile acids at the C7 position [12]. This means that the mouse liver is able to back-transform secondary into primary bile acids.

CYP2C70 knock-out and CYP2A12/CYP2C70 double knockout mouse models were generated in an attempt to humanize the murine bile acid pool composition [8,12]. Indeed, these mice exhibit a human-like bile acid pool with low concentrations of MCAs [8,12], but the accumulation of CDCA in CYP2C70 and CYP2A12/CYP2C70 knockout mice associates with chronic liver inflammation [8,12,13], questioning the relevance of such models to study liver diseases. Additional ‘humanization’ of the gut microbiota might be required to increase the translational relevance of the model.

## Bile acids in health and NAFLD

The array of bile acid species variably interact with receptors, resulting in diverse (patho)physiological outcomes [14]. Many receptors do accept bile acids as ligands but the nuclear Farnesoid X receptor (FXR) and the membrane Takeda G protein-coupled receptor 5 (TGR5) are specific for bile acids (Figure 2). FXR, expressed in enterocytes and hepatocytes, acts as a bile acid sensor and fine-tunes the bile acid pool size and composition. FXR activates a negative feedback response that represses intracellular bile acid uptake and synthesis, and promotes bile acid secretion. The activation of TGR5 also controls the delivery of bile acids to the gut through the regulation of gallbladder filling and bile flow [15].

Besides, bile acids diversely and competitively engage FXR and TGR5 receptors (Figure 2) to significantly impact on lipid and glucose homeostasis, energy expenditure, inflammation and fibrosis [16–19]. Hence, NAFLD is a disease in which it is anticipated that the modulation of bile acids might become a game changer. Along with gain- and loss-of-function animal models, studies using FXR, TGR5 and dual agonists support a beneficial effect in NAFLD and its dysmetabolic context [17,19,20]. However, agonists agents cause unwanted side effects that limit their clinical use [19,20]. Supplementation in bile acids at supraphysiological doses achieves protection against NAFLD (Table 1) but at the cost of adverse effects. Interestingly, intestinal FXR inhibition has also been reported to protect against glucose intolerance [17].

**Table 1:** Effects of bile acid administration in experimental models of NAFLD

Bile acid	Animal model	Dose	Duration	Administration route	Effects on NAFLD
**CA**	KK-A^y^ mice [36]	0.5%	8 weeks	Dietary	↘ Hepatic triglycerides
	*ob/ob* mice [37]	0.5%	4 weeks	Dietary	↘ Steatosis
**DCA**	HFD-fed *foz/foz* mice [38]	0.03%	12 weeks	Dietary	↘ Steatosis
		0.1%	12 weeks	Dietary	↘ Steatosis, ballooning and macrophage infiltration
**UDCA**	*ob/ob* mice [37]	0.5%	4 weeks	Dietary	↘ Steatosis
	HFHC fed-C57BL/6 [39]	120 mg/kg	4 weeks	Intragastric	↘ ALT and AST, unchanged NAS ↘ Inflammation and ballooning
	MCD-fed rats [40]	80 mg/kg	4 weeks	Intragastric	↘ ALT and AST ↘ Steatosis, inflammation and ballooning
	HFD-fed KK-A^y^ mice [41]	150 mg/kg	2–3 weeks	Oral gavage	↘ Hepatic triglycerides, no effect on histology

Abbreviations: HFD, high-fat diet; HFHC, high-fat high-cholesterol diet; MCD, methionine-/choline-deficient diet. Doses are expressed as bile acid concentration in the diet (w/w) or as daily dose in milligram per kilogram of body weight.

Table 2 summarizes data on bile acid pool in patients with NAFLD. In systemic blood, total bile acids were found higher in patients with biopsy-proven NAFLD compared with controls in 6/10 reports but unchanged in 3/10. When higher, total bile acids positively correlated with fibrosis severity [21]. Disrupted balance between primary bile acids (reflecting hepatic synthesis) and secondary bile acids (reflecting transformation by gut microbes) is a repeated finding, though the direction of the disturbance is inconstant. Different experimental conditions such as sampling site and timing, patients and controls characteristics, and methodology for bile acid analysis do not allow to define a bile acid signature in NAFLD patients.

**Table 2:** Studies reporting alterations of the bile acid pool size and composition in biopsy-proven NAFLD patients

Sampling site	Groups	Total bile acids	Bile acid composition
Liver	NASH vs HC	Higher [21]	Higher (T)CA, (G)CDCA and (T)DCA [21,22] Lower CA and GDCA [22] Higher conjugated CDCA/MCA ratio [23]
	NASH vs steatosis	Not available	Higher TDCA [22] Lower GDCA [22]
Systemic plasma or serum	Steatosis vs HC	Higher [23]	Higher TCA [24] No change [23]
	NASH vs HC	Higher [25–30] or unchanged [15,23,31]	Higher total primary BA [23,29,30,32] Lower total secondary BA [23,28,32] or higher total secondary BA [29,30] Higher total conjugated BA [25,28–30,32] Lower total unconjugated [15] Lower taurine to glycine conjugated BA ratio [28] Higher (G/T)CA, (G/T)CDCA, (G/T)UDCA and (G/T)DCA [23,24,27–30,32,33] and GHCA, HDCA, 3-oxo-DCA, iso-DCA [29] Lower LCA, 7-oxo-DCA [29], MCA [23], CA and DCA [15] Conjugated CDCA/MCA ratio positively correlated to steatosis, inflammation and ballooning [23] 7-oxo-DCA and 7-oxo-LCA positively correlate to NASH severity and ballooning [29] No change [31]
	NASH vs steatosis	Unchanged [15,23]	Lower total unconjugated BA [15] Higher total primary BA [23,32] Lower (G/T)CA, (G/T)DCA [15] and βMCA [23] Higher (G/T)CA and (G/T)UDCA [32]
	Fibrosis vs no fibrosis	Positively correlate to fibrosis [15]	Higher total primary BA [15] Lower unconjugated BA [15] Lower secondary/primary BA ratios [32,34] Conjugated CDCA/MCA ratio positively correlates to fibrosis [23]
Feces	Steatosis vs HC	Unchanged [26]	Higher CA [26]
	NASH vs HC	Higher [26]	Higher CA, CDCA [26] Lower LCA [26]
	NASH vs steatosis	Unchanged [26]	Higher GLCA and TLCA [26]
	Fibrosis vs no fibrosis	Higher in non-obese and lower in obese [35]	Higher CA, (G)CDCA, (G)UDCA in non-obese [35] Lower LCA and DCA in non-obese [35] Higher LCA and DCA in obese [35]

Abbreviation: HC, healthy control.

## Strategies to modulate the bile acid pool

Rather than bile acid supplementation or treatment with agonist or antagonist of bile acid receptors, tuning each of the determinant steps of the enterohepatic cycle (flags in Figure 1) might reshape the bile acid pool for therapeutic purposes.

### Flag 1: hepatic synthesis of bile acids

In hepatocytes, the bile acids taken up from the portal circulation add up to the newly synthesized ones. The resulting bile acid pool activates FXR, leading *in fine* to the repression of enzymes involved in bile acid synthesis (Figure 3). The dimerization of the FXR target small heterodimer protein (SHP) with transcription factors such as the liver receptor homolog 1 and the hepatocyte nuclear factor-4α represses the expression of CYP7A1 and CYP8B1 [22–24]. In ileal enterocytes, the absorbed bile acids control the release of fibroblast growth factor 19 (FGF19 or FGF15, the murine ortholog) into the portal circulation, which upon binding to the hepatic FGFR4-βKlotho complex represses hepatic CYP7A1 [24–27]. Thereby, FXR finely adjusts the bile acid synthesis in relation to the bile acid load entering the portal circulation.

The deletion of CYP7A1, the rate-limiting enzyme of the classical pathway for bile acid synthesis, reduces the bile acid pool size and up-regulates the enzymes of the alternative pathway [28]. This shifts the proportions of bile acids produced to a lower proportion of TCA and a higher proportion of TβMCA [28], as most of the CDCA synthesized through the alternative pathway is further converted into βMCA in mice (Figure 3). In the CYP7A1/CYP27A1 double knockout model, the bile acid pool size is drastically reduced but its composition remains unchanged [29]. This illustrates that hepatic bile acid synthesis determines in a large part the bile acid pool size and composition. FXR agonists and FGF19 analogs are thus perfect candidates to modulate bile acid synthesis (Figure 3).

Pharmacological FXR agonists, being bile acid analogs (obeticholic acid, OCA) or non-steroidal compounds, inhibit bile acid synthesis by acting at the hepatic and intestinal levels [22,25,30–36]. The demonstration that FXR agonists PX-102 in healthy subjects [32] or MET409 and Cilofexor in NASH patients [35,36] reduce bile acid synthesis, with no significant increase in circulating FGF19 concentration, indicates that the activation of hepatic FXR might inhibit bile acid synthesis independently of FGF19. On the contrary, the bile acid analog OCA dose-dependently increases FGF19 concentrations and, in parallel, decreases C4 concentrations in systemic blood of NAFLD subjects [33], suggesting a FGF19-dependent inhibition of bile acid synthesis. Fexaramine, a gut-restricted FXR agonist that does not activate hepatic FXR induces FGF15 production and portal secretion in mice with variable effect on hepatic bile acid synthesis and pool according to the studies [37–40]. Aldafermin (NGM282), a non-tumorigenic engineered analog of the gut hormone FGF19, suppresses the expression of CYP7A1 and CYP8B1 in mice [41,42] and hence, depletes the bile acid pool. The bile acid production relies mainly on the alternative pathway resulting in a bile acid pool mainly composed of TβMCA [41]. In NASH subjects, Aldafermin dose-dependently reduces total bile acids [43–45] and the proportion of 12α-hydroxylated bile acids in serum, signing the inhibition of the CYP8B1-dependent classical pathway [44].

The strategies to modulate the alternative pathway are less characterized. The rate-limiting step of the alternative pathway is the cholesterol being transported through the inner mitochondrial membrane by steroidogenic acute regulatory protein 1 (StarD1) [46]. Oxysterols generated by CYP27A1 activate liver X receptor and up-regulate StarD1 [2]. The balance between the classical and the alternative pathways might be influenced by environmental factors, as it will be discussed below. The promotion of the alternative pathway at the expense of the classical pathway would yield to intrahepatic accumulation of toxic oxysterols in humans, and/or to a more FXR-antagonistic Tα/βMCA-rich bile acid pool in mice.

### Flag 2: hepatic secretion of bile acids

BSEP is a major player for hepatocyte bile acid secretion (Figure 4). When absent or not functional, severe cholestasis occurs in humans. In mice, cholestasis is milder, as mouse hydrophilic tetra (3α-, 6α-, 7α- and 12α-) hydroxylated bile acids (Figure 2) are also excreted through the multidrug resistance protein 1a et 1b (MDR1a/1b) and the multidrug resistance associated-protein 2 (MRP2) [47–49]. While low in normal conditions, basolateral bile acid efflux through OSTα/β, MRP3 and MRP4 increases upon cholestasis causing bile acid spill over into the systemic blood [6]. Alternatively, a proportion of the bile acids secreted in bile canaliculi are taken up by the cholangiocytes that express ASBT and OSTα/β to return back to the hepatocytes; a process called the cholehepatic shunting that enhances bile secretion and flow [6].

The bile acid transporters in hepatocytes and enterocytes are under the regulation of FXR (Figure 4) [50]. FXR activation launches a coordinate response to increase export (BSEP, OSTα/β) and reduce import (NTCP, OATPs), and inhibit bile acid synthesis to protect the cells and the organism from bile acid overload. It will therefore be tempting to propose that increasing BSEP efficiency might confer hepatoprotection against raised bile acids.

### Flag 3: gut microbial transformations of bile acids

The complex community of microorganisms living in the gut interacts with bile acids [5,51]. The microbial conversion of primary bile acids into secondary bile acids is a key determinant of the bile acid pool composition (Figure 1). Germ-free mice have a poorly diverse bile acid pool only made of conjugated primary bile acids [52–54]. Similarly, treatment with antibiotics leads to depletion of secondary bile acids [53,54].

**Figure 5:**
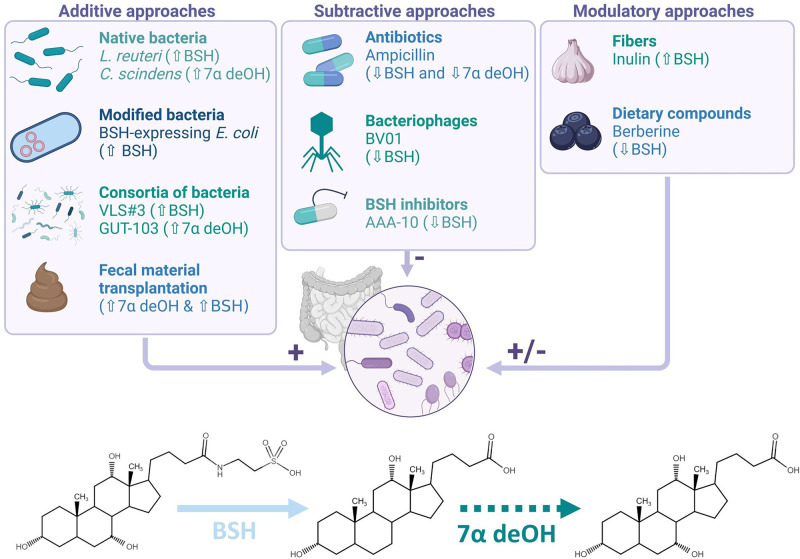
Different microbiota-based approaches to modulate the bile acid pool For each strategy, one example and its impact on the bile acid metabolism are mentioned. The BSH removes the glycine or the taurine from the primary bile acid (TCA represented) and allows the further conversion through the multistep 7α-dehydroxylation (7α deOH) into the secondary bile acid (DCA represented).

Bile salt hydrolase genes are widely distributed and highly abundant in gut bacterial and archaeal species [4,55]. Different BSH sequences yield enzymes with variable activities (optimum pH, substrate specificity) but all support the same reaction: the hydrolysis of the glycine or taurine moieties (Figure 5) [51,55,56]. Deconjugation by BSHs is a gateway step for subsequent transformations, amongst which dehydroxylation of unconjugated primary bile acids by the 7α-dehydroxylase is the most impactful on the bile acid pool composition [57]. This transformation converts CA into DCA and CDCA into LCA (Figure 5). Conversely to BSH activity, the 7α-dehydroxylation is a multistep enzymatic process carried by a small subset of low abundance bacterial strains [5]. Among the characterized strains are *Clostridium scindens* ATCC 35704 and VPI 12708, *Clostridium hylemonae* TN271, *Clostridium hiranonis* TO931, *Clostridium sordelii* VPI 9048, *Clostridium Leptum* VPI 10900 and *Extibacter muris* (exclusively in mice) [58,59], which are estimated to account for one millionth of the gut microbes in humans [57]. The bile acid inducible (*bai*) operon was characterized in *C. scindens* VPI 12708, in which the 7α-dehydroxylase activity is induced by CA [59]. The *bai* operon contains 8 genes coding for 7 enzymes (*baiB, baiCD, baiE, baiA2, baiF, baiH* and *baiI*) and one transporter (*baiG*) [57]. Recently, it has been shown that *Bacteroides vulgatus, Bifidobacterium adolescentis* and *Roseburia intestinalis* support CA 7α-dehydroxylation *in vitro* [60]. Apart from 7α-dehydroxylation, other transformations such as oxidation or epimerization further increase the diversity of the bile acid species. In humans, UDCA is formed through epimerization of CDCA by the gut bacteria and not synthesized in the liver as in mice. Besides, various bacteria are capable of generating bile acid isoforms such as iso-, 3-oxo-, allo-, 3-oxoallo- and isoallo-LCA and DCA [57,61–66]. Also, a microbial reconjugation to leucine, phenylalanine and tyrosine has been described [67]. This is an outline of the main modifications of bile acids by microbial enzymes; however, given the diversity of the gut microbial organisms, their isolated or synergistic activities, the number of sites on the sterol backbone available for modification and the variety of possible moieties, the potential diversity of the bile acid species is almost boundless. It is therefore of great interest to understand the impact on health of a change in bile acid metabolism in the gut, and thus in bile acid diversity.

#### Additive approaches

The administration of probiotics increases BSH activity [68,69]. Indeed, administration of *Lactobacillus acidophilus* and *Lactobacillus gasseri* with a high BSH activity to germ-free mice augmented the concentration of free bile acids [56]. Similar results were obtained in germ-free and conventionally raised mice with an engineered *Escherichia coli* in which BSH bacterial enzymes from *Lactobacillus salivarius* JCM1046 and *L. salivarius* UCC118 have been cloned [70]. Russel et al. even achieved a lifetime stable gut colonization in conventionally raised mice after a single administration of a native *E. coli* genetically modified to express a BSH [71]. Strikingly, three months after the single administration, secondary bile acids were higher and primary bile acids lower in feces of treated mice, reflecting the conversion of primary deconjugated bile acids into secondary bile acids [71]. Similar results were obtained with VSL#3, a probiotic mix containing *Lactobacillus, Bifidobacterium* and *Streptococcus* strains [72,73]. As a result of a low proportion of conjugated bile acids, the ileal active reabsorption of bile acids and production of FGF15 were reduced, thereby increasing fecal excretion and hepatic synthesis of bile acids [73]. In humans, microencapsulated *Lactobacillus reuteri* NCIMB 30242 (and even more if encapsulated in a delayed release capsule [74]) increased the intraluminal BSH activity and plasma concentrations of free bile acids, supporting efficient reabsorption of free bile acids [75,76]. Similar results were observed in obese subjects using *Bacillus subtilis* R0179 or *Bifidobacterium animalis subsp. lactis* B94 [69,77].

The administration of *Clostridium scindens* which has a high 7α-dehydroxylase activity [78] increases the concentrations of DCA and LCA in antibiotics-treated [79,80], in gnotobiotic (i.e. germ-free mice colonized with few identified bacterial strains) [81,82] and in specific pathogen free mice [81]. Similarly, the colonization of gnotobiotic mice by *Extibacter muris* increases the proportion of secondary bile acids, without changing the bile acid pool size [58]. Because the 7α-dehydroxylation of primary bile acids is a multistep pathway, the transfer of the *bai* operon in a chassis is more complex than for BSH. The colonization of germ-free mice with an engineered *Clostridium sporogenes* in which the enzymes determined as necessary and sufficient to carry out the 7α-dehydroxylation were cloned, led to the production of DCA, although substantially less than in mice colonized with *C. scindens* [57]. The colonization of a reduced-community gnotobiotic model with GUT-103, a consortium of 17 bacterial strains that includes *C. scindens*, was shown to increase deconjugation and the conversion of primary into secondary bile acids [83]. A another study showed that treatment with a consortium of four bacteria containing *C. scindens* was not more effective to modify the luminal concentration of DCA than *C. scindens* alone in antibiotics-treated mice [79]. In the context of *Clostridium difficile* infection, the administration of a consortium of five *Lactobacilli* and two *Bifidobacterium* strains to mice reduced the primary bile acids and increased the secondary bile acids in feces [84]. A combination of *C. scindens* and an engineered isoDCA-producing *Bacteroides fragilis* or *Bacteroides ovatus* was also used to induce the conversion of CA into DCA and then into isoDCA in germ-free mice [64].

The fecal bile acid pool of patients with recurrent *C. difficile* infection is characterized by nearly undetectable secondary bile acids, which in normal conditions limit the growth of the pathological strain [85–88]. In that particular context, fecal material transplantation (FMT) from healthy donors allows the expansion of bacteria with BSH and 7α-dehydroxylase activities [60] and increases the BSH genes and the metagenomic-predicted BSH and 7α-dehydroxylase activities [87]. As a result, the FMT increased the secondary bile acids DCA, LCA and isoDCA [88] and decreased TCA [87], to yield a bile acid pool similar to that of healthy donors. This has been confirmed by other studies [85–87]. Similar though less marked results were also obtained after oral administration of capsules containing freeze-dried donor fecal microbiota [89].

To summarize, these studies illustrate that adding one or more bacteria—either native or modified—effectively affects diverse microbial bile acid-metabolizing activities and consequently the bile acid pool. Additional studies are required to assess the effects on the bile acid pool in the pathophysiological contexts.

#### Subtractive approaches

In mice treated with antibiotics, deconjugation being suppressed, the bile acid pool is mainly composed of TCA and TβMCA [53,54,90]. The bile acid pool size is large because TβMCA, a potent FXR antagonist, maintains unrestrained bile acid synthesis [53,91]. In humans, antibiotic treatment drastically increases the proportion of CA without changing the total bile acids in feces [92,93].

Bacteriophages alter the abundance and activity of their bacterial host and thus modulate the gut microbiota in a more specific manner than antibiotics. The *Bacteroides* phage BV01 was shown to repress the tryptophan-rich sensory protein in *Bacteroides vulgatus* ATCC 8482 which, via an unknown mechanism, repressed bile acid deconjugation [94].

Inhibitors of bile acid deconjugation have been developed [95,96]. In conventionally raised mice, the pan-BSH inhibitors (AAA-2 and AAA-10) reduced the BSH activity and shifted the intestinal bile acid pool towards predominantly conjugated bile acids [95,96]. As further 7α-dehydroxylation relies on initial deconjugation, the concentrations of the secondary DCA and LCA were also reduced by the treatment with BSH inhibitors [95,96]. Similar to changes observed with antibiotics [53], the TβMCA concentration was higher in treated-mice, stimulating hepatic bile acid synthesis through CYP7A1 and increasing the concentrations of total and conjugated primary bile acids in intestinal content [95]. The circulating bile acid species and the gut microbial composition were not significantly modified by the treatment with AAA-10 [95].

Conversely to the additive approaches, the subtractive approaches mainly target the BSH activity and thus the deconjugation of bile acids from taurine or glycine. Antibiotics also significantly modulate the bile acid pool but at the cost of changing many others parameters. Their use is thereby complex for improving the understanding the impact of a change in microbial bile acid metabolism on health and diseases.

#### Modulatory approaches

A subtler but no less effective strategy to modify the gut microbiota is through modulatory approaches by prebiotics or dietary compounds [97,98]. Consumption of oligo-fructose enriched inulin increased the abundance of *Bifidobacterium* species and of *C. scindens* (7α-dehydroxylase activity) and decreased the abundance of *Bacteroides vulgatus* (BSH activity and putative 7α-dehydroxylase activity), but had no effect on the fecal bile acid pool in overweight children [99]. By changing gut microbial structure and function, oligofructose increased the conversion of primary TβMCA into ωMCA, HDCA and HCA, without changing the total bile acid concentration in portal blood of mice [100]. *In vitro*, the BSH activity of *Lactobacillus acidophilus* NCTC 1723 increased when incubated with inulin, while it decreased with lactulose or lactobionic acid [101], indicating that prebiotics can alter the capacity of microorganisms to deconjugate bile acids *in vitro*, but further studies are required to assess the effects *in vivo*.

In mice, berberine elevated total bile acids in feces [102–105]. While some studies showed that berberine did not affect the circulating and hepatic bile acid profiles [102], other reported that it elevated conjugated and primary bile acids [103–105] and reduced secondary bile acids [104]. Berberine changed the microbial community function and particularly, lowered the BSH activity [102,104,106]. Soybean protein also changed the gut microbiota composition and the caecal bile acid pool to higher proportion of secondary bile acids, without affecting fecal bile acid loss [107]. By inducing the expansion of *Bifidobacteria* and *Lactobacillus* genera, phenolic blueberry extract lowered the ratio of primary to secondary bile acids that was elevated by a HFD [108]. A component of Pu-erh tea, theabrownin also modulated the bile acid pool size and composition through suppression of microbes of the *Bacillus, Streptococcus, Clostridium* and *Bacteroides* genera that have a BSH activity and activation of the alternative bile acid synthetic pathway in mice and humans [109].

By affecting the gut microbial structure and function, modulatory approaches that include the use of prebiotics and dietary compounds also impact the metabolism, the composition and the signaling of bile acids. Nevertheless, the impact of these modulations on host physiology remains currently elusive and poorly characterized.

All these emphasize that the interactions between the food, the gut microorganisms and the bile acids produced by the host are essential and have a crucial role in shaping the bile acid pool. Thus, adjusting the composition and/or function of the microbial communities that inhabit our gut has a real influence on the bile acid pool size and composition. It is worth noting that the results were mostly obtained in preclinical studies and in reduced models such as gnotobiotic or antibiotic treated animals, representing an important limitation of the translation and the application to humans.

### Flag 4: intestinal reabsorption of bile acids

The reabsorption of bile acids from the gut lumen into the portal circulation occurs through passive diffusion or active transport that requires transporters (Figure 4) [6]. The importance of ABST as a gate for the recapture of bile acids into the portal circulation has been demonstrated using ABST knockout models [7]. ABST favors the transport of glycine and taurine conjugated bile acids over unconjugated bile acids [6]. When ABST is absent or inhibited, bile acids remain in the intestinal lumen and are more likely to undergo microbial transformations, promoting their reabsorption through passive diffusion for the most hydrophobic ones or their excretion in feces.

Hence, ABST inhibitor SC-435 and others increased fecal bile acid loss [110,111] and stimulated the classical pathway for hepatic bile acid synthesis through the FXR-FGF15 pathway, which caused relative reduction of MCAs [112]. Thus, ASBT inhibition renders the bile acid pool more hydrophobic. In humans, the inhibition of ABST by volixibat (SHP626), linerixibat (GSK2330672) or odevixibat (A4250) also increases fecal bile acid excretion and hepatic bile acid synthesis, hence decreasing the proportion of secondary bile acids [113–116]. The inhibition of OSTα-OSTβ by clofazimine concentrates bile acids in the enterocytes, leading to the activation of intestinal FXR, with no effect on the composition or size of the bile acid pool [117].

Bile acid sequestrants cholestyramine, colestimide or colesevelam are ion exchange resins that capture bile acids in the gut, preventing their reabsorption into the enterohepatic circulation and promoting fecal excretion [118]. The lower FXR-FGF15-SHP negative feedback then induces the synthesis of bile acids and lowers cholesterol in the liver [119–121].

ASBT inhibitors and bile acid sequestrants limit the absorption of bile acids into the portal circulation. Conversely, the overexpression of ABST, OSTα and/or OSTβ could result in the opposite outcome, i.e. increased bile acid enterohepatic recycling. Such approaches have not yet been tried by fear of hypercholesterolemia and/or hypercholanemia.

While conjugated bile acids are preferentially actively reabsorbed, unconjugated bile acids diffuse across cell membranes into the portal circulation according to their hydrophobicity [6]. Factors that affect the hydrophobicity are conjugation (e.g. to taurine and glycine) and the number of hydroxyl groups (Figure 2). Conjugation, deconjugation and other biotransformations by gut microorganisms (see Flag 3) can thereby significantly impact bile acid reabsorption. To illustrate, high BSH activity associates with high concentration of free bile acids in systemic blood, reflecting a high reabsorption [74–76]; while low BSH activity associates with elevated fecal bile acid loss [95,96,102]. Thus, changing the hydrophobicity of the bile acid pool by targeting the gut microbiota might increase or decrease passive bile acid flux across the intestinal epithelium.

### Flag 5: hepatic uptake of bile acids

The capture of bile acids in the hepatocytes through basolateral transporters, NTCP and OATPs, completes the enterohepatic bile acid cycle (Figures 1 and 4). NTCP preferentially captures conjugated bile acids and members of the OATP family unconjugated bile acids [122]. Subjects lacking NTCP and those treated with the NTCP inhibitor Myrcludex B have hypercholanemia [122–125]. In NTCP knockout mice, the phenotype is milder as conjugated bile acids are additionally transported in the hepatocytes by OATP1A1 and OATP1B2 [126–128]. This is also supported by the inhibition of the re-uptake of bile acids by Myrcludex B in OATP1A/1B knockout but not in WT mice [128–130].

As for intestinal bile acid reabsorption, strategies to increase bile acid capture by the hepatocytes might yield to a condensed enterohepatic bile acid pool due to enhanced bile acid signaling in the liver. Such an approach is of interest, as the combined activation of the bile acid-activated receptors TGR5 and FXR has been reported as protective against NASH progression [31,131–135].

#### Environmental factors

The bile acid pool constantly changes according to time and location along the enterohepatic cycle [136], identified as flags in Figure 1. The diet, biological clock, temperature, physical exercise, health status and genetics further confer a spatiotemporal bile acid signature to each individual. Here, we illustrate how some of these variables affect one or more flag(s) and how they might be tuned to reshape the bile acid pool.

Food products and dietary patterns modulate structural variants in the genome of gut bacteria, hence the bile acid pool [137]. High-fat and high-sucrose diet (HFHS) strikingly increases total bile acids and the proportion of conjugated primary bile acids in systemic blood and feces, through a modulation of the gut microbial communities and a reduction of the microbial BSH activity [138]. The concentration of the FXR antagonist TβMCA is elevated in HFHS-fed mice, resulting in enhanced hepatic bile acid synthesis through CYP7A1 [138]. Already high fat diet alone markedly alters the gut microbiota composition: it lowers the BSH gene expression [139] and increases the bile acid pool size and the concentration of TβMCA [140,141]. This was, however, not seen in animals fed a high fat, cholesterol and fructose diet [142,143]. In humans, a meat based-diet remodels the gut microbial communities and their metabolizing activities, already after a short period of time, with increased expression of microbial BSH and fecal concentration of secondary and of total bile acids [144]. Similarly, the circulating total, primary and glycine-conjugated bile acids are higher, while the fecal total, secondary and conjugated bile acids are lower in vegans compared with omnivorous [145]. This is just to cite a few of the reports supporting that dietary components shape bile acids by changing the gut microbiota [146–149].

Caloric restriction remodels the gut microbiota composition to lower BSH expression [139], resulting in reduced liver total bile acid concentrations in mice [139,150]. The resulting up-regulation of the classical pathway and repression of the alternative pathway for bile acid synthesis elevates the proportion of 12α-hydroxylated bile acids [139,150]. In humans, low caloric diets to obese subjects restructures the gut microbial community that associates with reduced total bile acids in systemic blood and feces [151–153].

In addition to being regulated by meals and synchronized by vesicular emptying, bile acids are dependent on the biological clock. CYP7A1 expression is regulated by the clock genes REV-ERBα and DBP [154,155] and FXR and SHP expressions have a circadian rhythm [156]. The expression of KLF15 in ileum cyclically inhibits the expression of FGF15 [157]. Short-term sleep disruption alters bile acid metabolism by suppressing clock genes as well as CYP7A1 in mice [158]. As a consequence, total bile acids are reduced in the liver and intestine of mice submitted to sleep disruption [158]. Similarly, chronic disruption of the circadian rhythm increased fecal CA and TCA, and decreased the gut microbial α-diversity in rats [159].

Comparing to thermoneutrality-housing [160], cold exposure stimulates bile acid synthesis through induction of CYP7A1 [161], CYP8B1, CYP7B1 and CYP27A1 [162,163]. This is accompanied by changes in the microbiota composition and functionality [162]. While Worthmann et al. reported a higher BSH activity [162], Zietak et al. hypothesized that the lower abundance of *Lactobacillus* after cold exposure contributes to the higher concentration of conjugated bile acids [162], as the members of the *Lactobacillus* genus have a high BSH capacity [164]. The higher levels of conjugated bile acids [163], such as Tα- and Tβ-MCAs that are FXR antagonists, would inhibit FXR signaling, increase the expression of enzymes for bile acid synthesis and consequently increase the bile acid pool size.

Physical activity changes the bile acid pool size and composition. Endurance exercise boosts fecal excretion and biliary secretion of bile acids in mice [165], with an even more pronounced effect in hypercholesterolemic mice [166]. The bile acid transporters OSTα and OSTβ in ileum and NTCP in liver are down-regulated in running versus sedentary animals [165], indicating a reduced bile acid reabsorption. Although the gene expression of CYP7A1, CYP8B1 and CYP27A1 is not affected by exercise in that study [165], CYP7A1 is up-regulated in trained mice in comparison to sedentary mice in another study [167]. In humans, circulating FGF19 and total bile acids are reduced after resistance exercise, while unchanged by endurance exercise [168].

## Application to NAFLD therapeutics

This section explores the biological tuning of bile acids along the enterohepatic cycle for therapeutic purposes in NAFLD.

Most studies report CYP7A1 up-regulation in NASH livers [169–171]. By contrast, the expressions of CYP8B1, CYP7B1 and CYP27A1 are uncertain [169–173]. StarD1 that mediates the trafficking of cholesterol for bile acid synthesis through the alternative pathway is up-regulated in NASH livers [46,174]. Overall, the data suggest a higher bile acid synthesis in NASH, although the total bile acid concentration was reported high in some [170,175–179] but not all the studies [21,169,171] (Table 2).

As bile acids are the main regulators of their own synthesis, detangling the cause and the consequence of bile acid perturbations observed in NAFLD is almost impossible. Nevertheless, FXR agonists and FGF19 analogs that target and dampen the classical pathway for bile acid synthesis have been quite uniformly reported to reduce liver fat content, NAFLD severity assessed by the NAFLD activity score and/or fibrosis in patients with NAFLD, even if often at the expense of negative side effects such as pruritus and higher atherosclerotic risk [20,35,45,180,181]. The beneficial effects cannot solely be attributed to indirect changes in the bile acid pool as FXR activation has numerous direct effects notably on glucose and lipid homeostasis, inflammation and fibrosis [17,19,24].

Advanced stages of NAFLD are sometimes associated with high hepatic bile acid levels (Table 2) [182]. Hence, boosting bile acid export out of the hepatocytes might confer hepatoprotection. This has however not been tested.

Targeting the gut microbiota and its functionality in NAFLD subjects is attractive, as gut dysbiosis contributes to NAFLD [183], with microbiome signatures being associated with the severity of NAFLD and associated fibrosis [184,185].

The administration of bacterial strains (either native or genetically modified), prebiotics and cold exposure have been shown to increase the BSH activity, the fecal excretion of unconjugated bile acids and the hepatic synthesis of bile acids from cholesterol. Higher BSH activity is thus logically accompanied by a reduction of cholesterolemia in different clinical studies on healthy and hypercholesterolemic subjects [75,76]. Cholesterol being a major contributor of lipotoxicity that drives NAFLD pathogenesis [186], increasing BSH activity might be effective in preventing NAFLD development. In mice, higher BSH activity and subsequent changes in bile acid pool are associated to reduced weight gain and liver triglycerides [70] and to improved glucose tolerance in both lean and obese mice [71]. By contrast, treatment with antibiotics, bacteriophage or BSH inhibitors and modification of the diet in terms of composition and of caloric load have been described to reduce the BSH activity. The inhibition of BSH by AAA-10 increased the abundance of conjugated bile acids in the gut, thereby preventing intestinal permeability and the subsequent development of steatohepatitis [187].

Currently, the impact of the elevation of specific bile acid species on NAFLD remains globally ignored. HCA and HDCA increase TGR5 activation, promote GLP-1 secretion and improve glucose tolerance and insulin sensitivity [100,188]. The administration of oligofructose elevates the concentration of HCA and HDCA in mice [100], but whether this contributes to oligofructose benefit is unknown. The administration of DCA protects from NASH [131]. Boosting the endogenous production of DCA such as shown in mice by the administration of *Ruminoccocus bromii* [185] might represent a therapeutic opportunity, not yet explored.

Dietary compounds also alleviate NAFLD, in a bile acid-dependent manner. In mice, camu camu protects from weight gain, fat accumulation and hepatic steatosis development [138], and berberine as well as silybin prevent from hepatic steatosis [103,189] and NASH [105]. The replacement of dairy protein in the regular HFD by soybean protein reduces weight gain and attenuates hepatic steatosis [107]. Rebalancing the bile acid pool through blueberry extract improves the metabolic syndrome [108]. Nevertheless, the impact of these modulations of the microbiota and consequently of the bile acid pool on NAFLD in humans remains elusive at the present time, and further investigations are then required.

Blocking the intestinal bile acid reabsorption by ASBT inhibitors and bile acid sequestrants leads to fecal bile acid excretion and subsequent cholesterol consumption to replenish the bile acid pool [112]. The inhibition of ASBT has been demonstrated to be beneficial for the treatment of NAFLD and associated dysmetabolism, as ASBT inhibitors lower hepatic and circulating cholesterol levels, improve glucose tolerance [110–112] and alleviate NAFLD severity in animals [112]. In humans, the ABST inhibitor linerixibat reduces fasting glycemia, insulinemia and improves glucose tolerance in patients with Type 2 diabetes [115]. However, in subjects with NASH, volixibat failed to reduce liver fat infiltration or hepatic injury [190]. In addition to their hypocholesterolemic effects [118,120,191], bile acid sequestrants have been shown to improve glycemic control through secretion of GLP-1, partially in a TGR5-dependent manner [121,191,192]. Colesevelam also protects against hepatic steatosis and fibrosis in mice [120]. In NASH subjects, colestimide promotes weight loss and reduces hepatic steatosis [193], but colesevelam was also reported to increase liver fat content [194]. Divergent data preclude any firm conclusion.

Blocking the hepatic recapture of bile acids is a strategy to increase the concentration of circulating bile acids and temporarily stimulate bile acid signaling in peripheral tissues. Notably, the activation of TGR5 and FXR in cells outside the enterohepatic cycle controls key metabolic processes such as food intake, energy expenditure and insulin sensitivity [16]. The postprandial elevation of serum bile acids induced by the pharmacological inhibition or genetic deletion of NTCP is accompanied by higher GLP-1 levels, reduction of diet-induced obesity and attenuation of liver fat infiltration in mice [130,195]. The protective effect of NTCP inhibition by Myrcludex B is partly due to a decreased intestinal fat absorption and greater fecal energy loss, and partly due to an increased brown adipose thermogenesis [130,195]. Currently, there is, to our knowledge, no data available on the promising effects of NTCP inhibition in NAFLD and metabolic diseases in humans.

## Conclusion and future perspectives

There is a plethora of strategies to tailor the bile acid pool, by tuning one or several key regulatory steps along the enterohepatic cycle or by adapting the environment. The strategies available as well as their application to NAFLD therapeutics were here screened and discussed. This review highlights the complex repercussions of modulating the size and composition of the bile acid pool, made of a mosaic of bile acid species and being the result of finely-tuned communications between the host tissues and the gut microorganisms. Although bile acid receptors FXR and TGR5 play pivotal roles in physiological homeostasis and in the treatment of pathophysiological conditions such as NAFLD, working upstream by reshaping the bile acid pool rather than directly targeting the receptors has been demonstrated effective and fruitful, consistent with the diverse and competitive binding of bile acids to receptors.

Before considering such strategy in the therapeutic armamentarium, some grey areas need clarification. First, appropriate and validated models to study both bile acids and NAFLD are required to enhance the understanding of bile acid-related mechanisms and to improve the translational relevance of the findings to humans. In this respect, there are new developments with generation of mice with CYP2C70 and CYP2A12 genes knockout, which have a ‘humanized’ bile acid pool. Once complemented by the ‘humanization’ of the gut microbiota and induction of NAFLD, they might provide data with high clinical relevance. Second, the intricate interactions between bile acids and the gut microorganisms are still to be dissected, though the task is demanding given the diversity of the gut microbial organisms and the multitude of bile acid species. We need to tackle this task to identify the microbial targets to skew bile acid metabolism and reshape a healthy bile acid pool in a tailored fashion and to test the effective consequences of microbiota-based interventions. Third, there is a strong rationale to modulate bile acid-receptors to treat NAFLD, though evidence of clinical efficacy is not convincing. Detangling the spatiotemporal complexity of the enterohepatic bile acid pool might help to better design new therapeutic strategies. There is still a long way to go, but the bile acid field clearly offers opportunities for breakthrough discoveries in personalized medicine for dysmetabolic disorders and NAFLD.

## Data Availability

Data sharing is not applicable to the paper.

## Abbreviations

ASBT: apical sodium-dependent bile salt transporter
3β-HSD: 3β-hydroxysteroid dehydrogenase
bai: bile acid inducible
BSEP: bile salt export pump
BSH: bile salt hydrolase
C4: 7α-hydroxy-4-cholesten-3-one
CA: cholic acid
CDCA: chenodeoxycholic acid
CYP27A1: sterol 27-hydroxylase
CYP7A1: cholesterol 7α-hydroxylase
CYP7B1: oxysterol 7α-hydroxylase
CYP8B1: sterol 12α-hydroxylase
DCA: deoxycholic acid
FABP6: fatty acid binding protein 6
FGF: fibroblast growth factor
FMT: fecal material transplantation
FXR: Farnesoid X receptor
HC: healthy controls
HCA: hyocholic acid
HDCA: hyodeoxycholic acid
HFHC: high-fat high-cholesterol diet
HFHS: high-fat and high-sucrose diet
HFD: high-fat diet
LCA: lithocholic acid
MCA: muricholic acid
MCD: methionine-choline deficient diet
MDR: multidrug resistance protein
MRP: multidrug resistance associated-protein
NAFLD: non-alcoholic fatty liver disease
NASH: non-alcoholic steatohepatitis
NTCP: sodium taurocholate co-transporting polypeptide
OATP: organic anion transporting polypeptide
OST: organic solute transporter
SHP: small heterodimer protein
StarD1: steroidogenic acute regulatory protein 1
TGR5: Takeda G protein-coupled receptor 5
UDCA: ursodeoxycholic acid
